# Entrepreneurial business start-ups and entrepreneurial failure: How to stand up after a fall?

**DOI:** 10.3389/fpsyg.2022.943328

**Published:** 2022-10-11

**Authors:** Lee-Yun Pan, I-Chih Tsai, Shu-Hui Popan, Shih-Chi Chang

**Affiliations:** ^1^Department of Business Administration, National Yunlin University of Science and Technology, Douliu, Taiwan; ^2^Department of Business Administration, National Changhua University of Education, Changhua City, Taiwan

**Keywords:** entrepreneur, entrepreneurship, entrepreneurial failure, bounce back, family support system

## Abstract

There are many reasons for entrepreneurs to start a business, but there is only a thin line between success and failure, and not everyone is willing to try to start a business again after encountering a failure. Therefore, it is worth exploring how start-up losers accumulate the energy of entrepreneurship and the reasons for starting a business again. In this study, the typical sampling method was adopted to select a suitable and representative case company entrepreneur for an in-depth interview. The results of this study revealed that in the process of the Entrepreneur starting a business three times, the Entrepreneur’s personal motivation and learning ability in the face of failure, coupled with family support, made the Entrepreneur willing to keep trying, even though he had to face the risk of repeated entrepreneurial failures, so that he could keep his positive energy on his entrepreneurial journey and eventually achieve a successful outcome.

## Introduction

Looking back at the past economic take-off period in Taiwan, small and medium-sized enterprises had a significant impact on the development of Taiwan’s economy, and the market development prospects were promising. People used rich and diverse channels to carry out a variety of entrepreneurial activities, which made the number of start-ups increase year by year. However, starting a business is not a simple matter. Nearly half of the small and medium-sized enterprises were in operation for less than 10 years, which means that new entrepreneurs continued to appear every year, while some business owners left due to business failure. Although most entrepreneurs hope to achieve success in the process of entrepreneurship, unfortunately, most of them end up in failure (Peng et al., 2010). Although most small and medium-sized enterprises have a small number of employees and a high degree of flexibility in operations that allow them to respond quickly to customer changes and needs, they often suffer from a lack of capital or human resources and are unable to cope with huge changes in the industry or the external environment because of their small size, resulting in failure.

Entrepreneurs start businesses for many reasons. Some people want to change their living conditions, some people identify new opportunities, and some people want to practice their goals or interests. Therefore, the discussion of entrepreneurship issues can be carried out from the aspects of entrepreneurial environment, conditions, results, and even entrepreneurial psychology (Lin and Wang, 2019). However, starting a business is not easy, and how to manage a business after a successful start-up is a major challenge. Often, there is a thin line between the success and failure of business start-ups, and the experience of failure has become a basic element of entrepreneurship (Lee et al., 2007, 2011). Still, failure experiences are not the main reason for entrepreneurial success (Yamakawa et al., 2010). Many past studies have explored the reasons for entrepreneurial failure or the entrepreneurial process for successful entrepreneurs. However, there are many entrepreneurs in the market who have stood up again after many failures. The main reasons for their standing up again may come from the entrepreneur’s personal leadership style, industry experience, or whether the entrepreneur has grasped the market demand. Also, whether the entrepreneur has important entrepreneurial resources is a relatively important advantage when starting a business. Finally, whether the entrepreneur chooses an environment and timing that is conducive to starting a business is an important factor. No entrepreneur can complete the process of starting a business alone, and they must interact with other individuals in society to find or create possible opportunities for cooperation (Steyaert and Katz, 2004; Eggers and Song, 2015). However, the entrepreneur’s past entrepreneurial achievements will affect the subsequent crowdfunding results (Soublière and Gehman, 2020).

Entrepreneurship is fascinating because of the achievements and gains brought by the success of entrepreneurship. Therefore, although starting a new business takes on many risks, including capital, changes in the industrial environment, and lack of experience, entrepreneurship is still the priority of most people. It is worth discussing how those people who have failed in starting a business accumulate the energy for entrepreneurship again and the reasons that promote entrepreneurs to start again and achieve success, as the greatest ability of entrepreneurs is to acquire and learn various skills from different experiences of failures (Yamakawa et al., 2010; Peng et al., 2014). Thus, in addition to studying the success factors of entrepreneurship, the reasons for failures should also be studied, as the new knowledge and skills gained can inject new opportunities for entrepreneurship (Minniti and Bygrave, 2001; Baron, 2004). This study aimed to understand how entrepreneurs treat the reasons for their failures in entrepreneurship, how they respond to setbacks, and how they adjust themselves and be willing to embark on the road of entrepreneurship again. Therefore, the research purposes of this study were to:

1. Understand the entrepreneurial process of entrepreneurs.
2. Understand the reasons for starting a business again after an entrepreneurship failure.
3. Understand the business leadership guidelines gained after a successful entrepreneurship experience.

## Literature review

### Entrepreneur theory

Entrepreneurship refers to starting a new business (Low and MacMillan, 1988). The entrepreneurial process can occur at any point in time (Liñán and Chen, 2009), so the entrepreneur’s intention in the first step is to create a long-term process and evolution for a new business (Lee and Wong, 2004). Entrepreneurs’ intentional execution of entrepreneurial behavior is a necessary beginning (Fayolle et al., 2006). Entrepreneurship is the creation of new services, technologies, and products based on the entrepreneur’s ideas or concepts, which serves as the foundation of the business. The essence of entrepreneurship comes from innovation, such as the creation of new products or new services. Therefore, innovation is the reorganization of resources by enterprises to meet the needs of the market in innovative ways, and it is the origin of gaining profit. Entrepreneurs provide customers with products and services of different value *via* various combinations of innovations (Schumpeter, 1934). Continuous entrepreneurs are an important driving force for national economic growth (Plehn-Dujowich, 2010).

The actual classifications and definitions of entrepreneurship are various and complex. Scholars have given different classification structures to discuss entrepreneurship according to different aspects. Shane and Venkataraman (2000) pointed out the conditions that entrepreneurship should have included the source of opportunities for entrepreneurship, the process of entrepreneurs discovering opportunities, entrepreneurship evaluations, and taking entrepreneurial action. Ozgen and Baron (2007) considered entrepreneurship to be multi-faceted, as pointed out in research on entrepreneurship from a gender perspective (Langowitz and Minniti, 2007) or the exploration of entrepreneurship from an international perspective (Terjesen et al., 2013). However, different industry life cycles will also affect entrepreneurial opportunities. In the emerging stage of the industry, the new industry has just started. It experiences great variability, uncertain operating periods, and immature technologies, as well as a large development space and a small number of existing companies, giving this market development potential for entrepreneurship. In the growing stage of the industry, the industry has existed for many years and there is a need for technical breakthroughs and transformations, but the existing companies have a fixed industrial scale and stable profit models. Therefore, the upstream, middle, and downstream manufacturers of the industry need to constantly think of innovative activities to promote the growth of the industry and create entrepreneurial opportunities. In the mature stage of the industry, the industry gradually declines. The market grows slowly, the demand tends to be stable, market compression leads to fierce competition within the industry, and the opportunity for new entrepreneurship is low. Unless there are breakthrough innovative technologies, this stage is not suitable for entrepreneurship. In the technology change stage of the industry, some entrepreneurs develop new technologies, products, raw materials, and products to replace the existing industrial technologies and enter the next industrial life cycle (Romanelli, 1989).

Entrepreneurship research has a cross-disciplinary nature, covers a wide range, and has neither a clear scope nor a specific theoretical framework. Therefore, many other theoretical foundations from different studies are often used to explain entrepreneurship research, such as social cognitive theory (Wang et al., 2019), social network theory (Chen et al., 2018), and resource-based theory (Kellermanns et al., 2016). In current entrepreneurship research theories, many scholars have proposed analytical concepts from different perspectives, but there is no integrated framework to list all the problems encountered in entrepreneurship. The field of entrepreneurship is complex and changeable, and a conclusion cannot be made through the study of a single dimension. Froese (2013) pointed out that the vision and motivation proposed by entrepreneurs can directly predict the possibility of an enterprise’s growth. To sum up the concept of entrepreneurship, entrepreneurship starts with the entrepreneur identifying new opportunities. Once the opportunity is found to have investment value, it is necessary to expand resources for execution. When the invested resources are effective, it represents a positive effect on entrepreneurial performance.

### Entrepreneurship

The uncertainty and economic returns brought by entrepreneurship are greater than the risks involved in employment, because the results of entrepreneurial efforts are unpredictable, and it is not the case that hard work will definitely bring success. Past research has shown that when individuals have a greater willingness to take the risks brought by a business, they will be more willing to start a business (Barbosa et al., 2007). That is to say, entrepreneurs are more willing to take risks than others (Stewart and Roth, 2001), and entrepreneurs with a stronger risk-taking tendency will pursue entrepreneurial opportunities with a higher risk (Forlani and Mullins, 2000; Mullins and Forlani, 2005).

Schumpeter (1934) pointed out that entrepreneurship is a kind of economic value creation, and it is the process of creating value, through which individuals or organizations carry out a series of innovative and creative activities and take advantage of opportunities. Miller (1983) mentioned that the entrepreneurial orientation can be discussed from the aspects of innovativeness, risk taking, and the proactiveness of management. Lumpkin and Dess (1996) further divided entrepreneurial orientation into five measurement dimensions for discussion, namely: innovativeness, risk taking, proactiveness, autonomy, and competitive aggressiveness. Innovativeness means that entrepreneurship should be based on targeted innovation to open new value through the combination of resources and the development of new products or services to establish the profitability of the entrepreneurial organization. Risk taking means that the entrepreneur must have an entrepreneurial spirit as well as risk-taking and problem-solving abilities. Proactiveness represents the entrepreneur’s instinctive ability to identify opportunities, as the ability to identify opportunities is an important foundation leading to entrepreneurial action. Finally, autonomy and competitive aggressiveness refer to the ability of the entrepreneur or group to improve and motivate themselves and have a positive attitude toward challenging competitors and persisting (Dess et al., 1997; Shane and Venkataraman, 2000).

Entrepreneurship is the core of an entrepreneur or entrepreneurial team and the driving force for the growth of an organization or enterprise, so it is an important factor affecting the operation of an enterprise. Schumpeter (1934) suggested that entrepreneurial spirit is a condition required to promote resource reorganization, and it is an inherent ability of an entrepreneurial team. Entrepreneurship is a concept of seeking innovation and pursuing change, as well as the behavior of creating and looking for opportunities. The biggest purpose of entrepreneurship is to re-create value and shape new needs, and entrepreneurship represents that the team has the ability to gain insight into opportunities, integrate resources, take risks and reshape values. If an entrepreneur or enterprise exhibits a positive entrepreneurial spirit, it will have a certain positive impact on the growth of the enterprise. Past research has shown that when an enterprise exhibits a high degree of entrepreneurship—that is, when it actively carries out various innovations for products, marketing, and markets, it can improve the operating performance of the enterprise; on the contrary, conservative action or inaction may not improve the performance of the enterprise. As mentioned above, if entrepreneurship is the key to entrepreneurial success, it means that entrepreneurship has a certain influence on entrepreneurial performance. Entrepreneurship is the introspective ability of entrepreneurs and entrepreneurial teams, which helps to motivate entrepreneurs to seek breakthroughs and innovations.

### Entrepreneurial failure

The success of past entrepreneurial experiences is a factor that affects whether entrepreneurs decide to start a new business again (Hsu et al., 2017). Every entrepreneurial failure affects the morale and sanity of the entrepreneurs (Nefzi, 2018), and negative emotional reactions, such as sadness, will deplete the entrepreneur’s learning ability (Shepherd, 2003). Therefore, experiencing many failures is not an effective way to improve experiences, and the accumulation of too much sadness will further hinder learning from failure experiences. Entrepreneurship is a way of self-fulfillment for many people. The rise of an entrepreneurial culture brings more and more entrepreneurs, but some failed entrepreneurs may be arrogant and unable to continue to learn and move forward (Hayward et al., 2006). Although entrepreneurs may face failure, they usually have enthusiasm and perseverance for entrepreneurship, so they keep moving forward (Chen et al., 2009). Many entrepreneurs have continuous and repeated entrepreneurial failures, yet they keep trying until they succeed (Flores and Blackburn, 2006; Hayward et al., 2006; Cardon et al., 2010). Hopp and Stephan (2012) and McGee et al. (2009) found that there is a correlation between entrepreneur self-efficacy and entrepreneurial motivation. Hsu et al. (2017) suggested that even if failures weaken the entrepreneurs’ self-efficacy, there are still many failed entrepreneurs who are willing to get back on the journey of entrepreneurship.

Many entrepreneurs are unable to recover after experiencing an entrepreneurial failure, thinking that they are not suitable for the industry, lack abilities (such as analysis abilities, learning abilities, or leadership abilities), are unable to affirm themselves, unwilling to take the risk of failure, lack entrepreneurial motivation and resources, and are unwilling to try again (Seligman, 2011). However, there are also entrepreneurs with strong psychological qualities as well as extraordinary will and desire, who are willing to continue to take risks, improve their own ability to strengthen the energy of entrepreneurship, shorten the time for entrepreneurial exploration, and are successful in entrepreneurship after many attempts. Entrepreneurs who have experienced a failure and re-opened in the early stage of the business have a low failure rate in future (Choi and Shanley, 2000). The reason may be that the entrepreneur has acquired certain professional knowledge, has accumulated the ability to judge things from the past failure experience, and has gained a relatively good understanding of risk assessment for a new start-up, giving a higher ability to solve problems as compared with the initial entrepreneurship. Entrepreneurs’ entrepreneurial experience has been explored in many previous studies, such as the cognitive impact of differences in failure on the generation of motivation to exit entrepreneurship (Ucbasaran et al., 2010, 2013). Surveys conducted on entrepreneurs with rich entrepreneurial experience regarding the quality and quantity of entrepreneurial experience (for example, with or without entrepreneurial experience) revealed that no matter what the quality of entrepreneurship was, past entrepreneurial experiences are helpful for future entrepreneurship (Zhao et al., 2005; Fitzsimmons and Douglas, 2011). Moreover, these experiences are also helpful for the performance of a new start-up after an entrepreneurial failure (Yamakawa et al., 2015).

Societal perceptions of entrepreneurial failure are significant (Baumol, 1993; Peng et al., 2009), and this social awareness also influences entrepreneurs’ perceptions of the reasons for failure and whether they want to continue entrepreneurship (Cardon et al., 2010). Therefore, entrepreneurs must have a strong psychological quality to start a business again (Hsu et al., 2017); they should be able to overcome bad memories of the past and criticism from others and have a high degree of patience to start a business again and endure the loneliness during the process of entrepreneurship. Since there is still a high risk of failure when starting a business again, only those with strong entrepreneurial motivations are more likely to be willing to take high-risk failures and enjoy the difficult process of entrepreneurship.

### Motivations for new business creation

As entrepreneurial risks are very high, how do entrepreneurs bounce back after entrepreneurial failures? Past research on entrepreneurs mostly explored the sources of their motivations, meaning why entrepreneurs are willing to accept entrepreneurial challenges and take action to start a new business again. The sources include entrepreneurs’ personality traits (Leutner et al., 2014; Kerr et al., 2018; Presenza et al., 2020; Vandor, 2021), age (Lin and Wang, 2019), self-efficacy, and resilience (McGee et al., 2009; Hopp and Stephan, 2012; Bullough et al., 2014; Maroor et al., 2018; Lafuente et al., 2019). There are also related studies on the effect of an entrepreneur’s ability to learn from failures, and then, start a new business in future, such as Plehn-Dujowich (2010), Yamakawa et al. (2015), and Soublière and Gehman (2020).

In addition to personal characteristics and cumulative ability, the impact of the cost of past failures on future re-entrepreneurship endeavors is also the main topic of discussion in this study (Eggers and Song, 2015; Nefzi, 2018). Generally speaking, while an entrepreneur’s failure in previous entrepreneurship can trigger his or her willingness to take on the next entrepreneurial venture (Sarasvathy et al., 2013), it is subject to varying levels of stigma, such as a sense of shame (Begley and Tan, 2001; Gratzer, 2001; Singh et al., 2015) or a huge debt (Amankwah-Amoah et al., 2021), which will hinder the motivation of entrepreneurs to start a new business. Singh et al. (2015) pointed out that entrepreneurs who have failed will eventually be able to develop a new career if they can overcome stigma and treat failures in a positive manner.

The educational level of entrepreneurs also affects whether they are willing to start a business again after an entrepreneurial failure. The results of Amaral et al. (2011) showed that people with higher education are often reluctant to start a business again after an entrepreneurial failure; however, spiritual faith can help entrepreneurs who have failed to look at their failures in a positive light and make them willing to start over (Singh et al., 2016). Specifically, it is beneficial to understand the positive factors that make entrepreneurs who experience start-up failures be willing to try again. Therefore, further research is needed to help clarify how entrepreneurs who have failed deal with their failures, and the heterogeneity experienced by entrepreneurs who have failed will likely influence their motivation to bounce back. The above-mentioned factors that affect the motivations for re-entrepreneurship focus on what entrepreneurs must change regarding their inner thoughts, while external factors beyond their control often discourage entrepreneurs who have failed to bounce back; for example, starting a business after an entrepreneurial failure will be affected by the bankruptcy policy where the company is located. Stringent bankruptcy policies will enhance entrepreneurs’ motivation to start again (Damaraju et al., 2021). In summary, while research has provided some knowledge regarding why entrepreneurs who have failed are willing to try again, more research is needed to understand whether there are any other influencing factors.

## Research methodology

### Research design

The purpose of this study was to explore the factors that cause entrepreneurs to start a new business after entrepreneurial failures. During the research process, it was necessary to deeply understand the entire entrepreneurial process of the Entrepreneur of the case company and the development background of each stage of the business after each entrepreneurial failure, the reasons for the establishment of another start-up, and the company guidelines after the successful start-up. Therefore, an interpretive case study based on in-depth interviews with company personnel was used for the exploration.

### Case collection

The typical sampling method was adopted by this study to select a suitable and representative case company Entrepreneur for in-depth interviews. In order to comply with the theoretical sampling principle, the subject selection needed to be consistent with the concept of starting a business again after an entrepreneurial failure. Therefore, the entrepreneur of a company who had succeeded after three unsuccessful entrepreneurial attempts was selected.

The case selected for this study was HD Biotechnology Co., Ltd., which mainly manufactures raw feed materials for the animal husbandry industry and is engaged in processes from pig breeding to food processing. The company integrates the upper, middle, and lower reaches of the industrial chain to provide customized feed mixing services, and specializes in consulting and analysis on raising pigs, food processing, and other knowledge to meet the needs of customers.

There were three reasons for choosing HD Biotechnology Co., Ltd. First, the entrepreneur of the company experienced three entrepreneurial journeys; second, the entrepreneur had numerous reasons for starting a business again after many entrepreneurial failures; third, the factors of the final entrepreneurial success after three entrepreneurial failures and the approaches led to a viable business.

### In-depth interviews

#### Interviews

The researcher compiled a semi-structured interview questionnaire based on the research purpose and literature review, mainly to understand the reasons and motivations of the entrepreneur of the case company in this study. The interview outline used problem clarification, problem analysis, and problem countermeasures as the main axes. The design outline of the interview questionnaire was divided into three categories, including (1) the motivation for entrepreneurship; (2) the mental journey of entrepreneurship; and (3) the reasons for starting a business again after an entrepreneurial failure. Table 1 lists the relevant data from the interview questionnaire.

**Table 1:** Interview subject and interview question design used in this study.

Interview subject	Founder of HD Biotechnology Co., Ltd.
Background experience	The Entrepreneur graduated from the Department of Animal Husbandry and Veterinary Medicine and used to work as a veterinary administrator on a livestock farm
Interview questions	Please explain the opportunity or situation under which you came up with the idea of entrepreneurship? Please share your mental journey of starting a business, including the first entrepreneurial success, and how to deal with crises after the entrepreneurial failure? What motivated you to start a business again after facing many entrepreneurial failures? What personality traits do you think an entrepreneur must have in order to accept challenges endlessly and become more courageous? In addition to the entrepreneur’s own ability, what other conditions for support (including family, employees, environment, and funds) are needed for entrepreneurship?

#### Observations

The observational method was adopted by this study to collect data on company-related operations, product profiles, and the entrepreneur’s organizational interaction atmosphere, and the data collected through the observational method were incorporated into the primary data of this study.

#### Documents and triangulation

In the process of collecting data on the process of starting a business again after an entrepreneurial failure, this study collected company operation records, data on award-winning deeds, and other documents. The above data were not only used as the background information of the case but were also compared and analyzed with the interview content and the data obtained from the observation to achieve the verification of triangulation. Data quality was ensured by checking different data sources, converting the audio files obtained from the interviews into text files and comparing them with the interviewer’s notes, and finally, carrying out verification based on triangulation analysis. All data analysis results were discussed and confirmed again by the researchers, and the phased research results were also checked by the entrepreneur of the case company to clarify the correctness of the content, thereby ensuring the quality of the data and the internal reliability of the research.

## Analysis and results

The findings of this study were divided into three parts. The first was the entrepreneurial process of the case, the second was the reasons for trying again to start a business, and the third was the operational guidelines after the entrepreneurial success.

### Entrepreneurial process

The entrepreneur had a background in agriculture, industry, animal husbandry, and veterinary medicine. Due to his professional skills in animal husbandry, disease, and breeding, he was engaged in related industries. Therefore, he had relevant knowledge on and contacts in animal husbandry, which gave him the opportunity for his first start-up. At that time, together with his partners, he was prepared to set up a trading company to import feed. Because the entrepreneur could grasp the market development needs, together with his professional knowledge and skills as well as the accumulation of business contacts, the business operated better than expected and enjoyed rapid growth at the beginning. Unfortunately, the hoof-and-mouth disease outbreak in Taiwan in 1997 prompted consumers’ doubts about meat products, which greatly affected farmers and the meat market, and the feed industry was not spared either. However, the double blow of the shrinking market and the partner running away with the company’s money did not defeat the entrepreneur’s determination and perseverance to start a business. He decided to start a business for the second time in November 1997 and began to assemble machinery and equipment to build a factory and resume the old business. Unexpectedly, a major fire occurred in the factory in 2008. After a period of reorganization, the entrepreneur resolutely embarked on a third entrepreneurial journey. This challenge allowed the entrepreneur to successfully start a business and secure a firm position in the industry, as he had learned from the three failures in the past and had gained the ability to deal with crises quickly. Table 2 lists the three-time entrepreneurial processes of the subject entrepreneur of this study.

**Table 2:** The three-time entrepreneurial processes of the subject entrepreneur.

Entrepreneurial stage	Entrepreneurial motivation	Entrepreneurial achievements	Reasons for entrepreneurial failure
First entrepreneurial process	Considering future financial expenditures at home	Mastering professional skills and contacts, and achieving good results from the first start-up	Hit hard by the hoof-and-mouth epidemic in 1997 Runaway partner
Second entrepreneurial process	Huge debt pressure	Self-owned pig farms used as breeding demonstration farms and rebounding pig prices	Fire in the factory in 2008
Third entrepreneurial process	Gratitude to family and staff for working together to get the company back in business	Resumption of all operations of the company within half a year and launch of food processing products and its own brand: Ease Kitchen	

### Reasons for re-entrepreneurship

This study adopted the viewpoint of Lumpkin and Dess (1996) and divided entrepreneurial orientation into five dimensions: innovativeness, risk taking, proactiveness, autonomy, and competitive aggressiveness. Besides these dimensions, the work/family border theory perspective was added to explore the factors of the three re-entrepreneurial processes of the research subject.

#### Innovativeness

Innovativeness refers to entrepreneurs seeking to create goals and unlock new value through resource combinations, products, or service development. During the interview, the subject Entrepreneur mentioned:

*“The first time I started my business was when I saw that Taiwan’s animal husbandry area was mainly in the south of the central region, which is the largest and most promising market in Taiwan. Therefore, I chose to start the import and manufacturing business of feed raw materials in the central region.”*

*“The second time I started my business, I found that the hoof-and-mouth disease epidemic was gradually slowing down, and the market demand for edible pigs had increased greatly. I not only decided to invest in the original feed manufacturing industry but also established a pig farm and actively sought government certification to become a demonstration breeding vendor of a self-owned pigsty.”*

*“The third time I started a business was because I saw endless cases of ‘black-hearted food’ in Taiwan, coupled with the improvement of people’s quality, as more and more attention was paid to food safety. So, a food processing factory was established to actively promote food safety and one-stop farming as business philosophies, and a self-contained brand, Ease Kitchen, was built.*

The entrepreneur had three entrepreneurial failures and started all over again because he had a strong entrepreneurial spirit. He had not only been deeply involved in the industry for many years but also constantly understood the needs of the market environment, which allowed new products and services to be created and new wealth to be developed. He had the spirit of innovation and the creation of new ideas and practices, and he succeeded and established a significant position in the industry through his innovativeness.

#### Risk taking

Entrepreneurs must have the willingness to invest important resources in opportunities with high uncertainty; that is, during the decision-making process, entrepreneurs must be willing to accept the possibility of more losses in exchange for higher potential rewards. The entrepreneur of the case company in this study mentioned:

*“The first time I started a business was because I had considered the future economic expenditure of my family. It is difficult for an ordinary employee of a company to cover all the expenses of a family, so I resolutely embarked on the entrepreneurial journey.”*

*“The second time I started a business was because the partners of the company ran away with the money and left a huge debt. I had to bear the pressure of repaying all the debts, after which I started a business again, because I had no money to worry about, so I could give it a shot.”*

*“The third time I started a business was because of the damage to the factory caused by a natural disaster. As the person in charge of the company, I must let my employees have a secure job and make a living. Therefore, I started the business again to provide a stable working environment for my employees, who had supported me for many years.”*

Regarding risk taking, the most important factor of the entrepreneur repeatedly starting a business was having a courageous and adventurous spirit, the willingness to take risks, and a sense of responsibility. He desperately invested all that he had in exchange for the chance of success.

#### Proactiveness

Proactiveness refers to the entrepreneur’s ability to identify opportunities. The ability to identify opportunities is an important basis for entrepreneurial action, just as the first-mover advantage comes from exploiting the market and taking advantage of its asymmetry to obtain excess profits. The entrepreneur of the case company in this study mentioned:

*“I started my business for the first time because I saw the vigorous development of animal husbandry in Taiwan. Most of the industrial chain was concentrated in the south of the central region, so I chose to set up a raw feed material import and feed manufacturing factory in Yunlin.”*

*“The second time I started my business was because the hoof-and-mouth disease epidemic in Taiwan was slowing down, and the market demand for edible pigs was increasing day by day. Therefore, I decided to resume the old business and start the raw feed materials import and manufacturing business, and I established my own pig farm to increase the company’s products and get more profit.”*

*“The third time I started my business was because of the outbreak of food safety issues in Taiwan in 2011. The issue of ‘black-hearted food’ has attracted great attention from the media and society. The public has gradually begun to pay attention to issues such as food manufacturing sources, production methods, and additives, so I decided to invest in a food processing business to provide consumers with knowledge about the process of pig raising, slaughtering, and food processing, to alleviate consumers’ doubts about food safety.”*

The entrepreneurial spirit of the entrepreneur is nothing more than the ability to dominate opportunities and the ability to become the first operator to enter the market or to bring new stimuli to an existing market, which is the best proof of proactiveness.

#### Autonomy

In the process of seeking opportunities, entrepreneurs show a degree of willingness to improve themselves and motivate themselves, which is similar to the process of taking a concept to an idea and to the actual presentation. The entrepreneur of the case mentioned:

*“I think an entrepreneur must have a sense of responsibility and a persevering character, be willing to take care of others, help others, and have the spirit of taking risks in adversity. Whenever I encounter different setbacks, I take every test as the cornerstone of success and the nutrients that nourish our growth. There is no difficulty that cannot be overcome, and we only need to conquer ourselves. The more setbacks you overcome, the more you will be able to climb to a higher height.”*

With a positive heart, the entrepreneur actively sought the opportunity to stand up again after each business failure. This sense of responsibility motivated him to continue to start a business and have the courage to take on every hardship.

#### Competitive aggressiveness

Entrepreneurs have the attitude of challenging competitors and a high degree of persistence in each re-start. During the discussion, the entrepreneur mentioned:

*“The first time I started my business was because I was familiar with the industry myself. When Taiwan’s economy took off, the domestic market for edible meat increased greatly, and most of the industrial chain was concentrated in the south of the central region. Compared with absolute beginners, I had the blessings of relevant industry experience and geographical relationships, which made it more clear for me to invest in the import of raw feed materials and feed manufacturing.”*

*“The second time I started my business was because I saw that consumer demand for edible pork was increasing, so the business was oriented to customer needs. I would provide what customers need. We not only produced hoofed animal feed but also raised our own pigs and expanded our product projects to increase our enterprise competitiveness and create added value.”*

*“The third time I started a business was because Taiwan’s industrial development was mainly based on the OEM industry. For the first two start-ups, I did not dare to create a brand by myself, because I did not know how to market my own brand. However, in 2011, food safety issues broke out in Taiwan one after another. The food safety awareness of consumers was on the rise, so we decided to enter the food processing market, to check the source of food for consumers. We provide consumers with a transparent production process, including pig feed preparation, the feeding process, slaughtering, and food processing, from beginning to end, to create the brand of ‘Ease Kitchen’. We manufacture products with the concept of making consumers feel at ease to eat and have gained differentiated advantages with competitors in the same industry.”*

The entrepreneur started with feed manufacturing and moved to setting up farms and finally establishing his own brand. The three start-ups along the way injected new transformation products into each entrepreneurship, and the third start-up created a new brand value for the enterprise, which was also the biggest difference from the existing competitors in the industry. The entrepreneur’s ambition and hard work could be seen in every re-start.

#### Support from important family members

Based on the work/family border theory by Clark (2000), people are border-crossers between the work and family fields every day. This conceptual framework attempts to predict when conflicts between work and family will occur and how border-crossers can achieve a balance. Therefore, this theoretical basis was used to explore the influence of the family support provided by important family members in the work field and the family field on the entrepreneur’s re-entrepreneurship. The support from family members was an important source of motivation for the research subject that encouraged him to stand up again after facing repeated entrepreneurial failures. During the discussion, the entrepreneur mentioned:

*“The first entrepreneurial failure was because my partner ran away with the money, which caused me to owe a large amount of debt. In addition to my own expenses and mortgage, I also had to repay the bank interest of NTD 300,000 a month. In the face of the bank’s urging, when my house in my hometown was foreclosed, my wife was still persistent and constantly encouraged me by saying:* ‘*Even if the money is gone, at least our home is still there*.’ *This sentence woke me up from the failure, as it turned out that my family still needed me, and I had to cheer up.”*

*“The second time I failed to start a business was because of a fire in the factory. Since the imported raw materials were paid in cash and the completed orders and machines were also stored in the factory, all the tools for making money disappeared overnight. This fire made the company bear the burden of a huge debt again. Bad luck had come again. After the fire, my wife and employees came back and forth every day to transport the raw materials that could still be used to the outside of the factory for sorting, hoping to reduce the company’s burden to some extent. I watched the spontaneous actions and attitudes of my wife and colleagues and was moved deeply.”*

*“Family support is the biggest motivation for me to stand up again after repeated failures. Without the company of my wife and children, I would not have succeeded in starting a business today. After every entrepreneurial failure, my wife will always say*: ‘*It’s okay, let me accompany you to undertake the burden*.’ *This sentence makes me both annoyed and gratified. I have re-started my business three times, and this sentence has been deeply echoed in my mind for a long time. It is my backing and my strength to start again. I believe family support is the main reason why I’ve been able to get up again and again from failure and succeed at last.”*

Every successful entrepreneur has a strong motivation to make them enter the road of entrepreneurship again, just like the family support of the entrepreneur in this study, which was the best backing for him to move forward without fear.

### Guidelines for company operation after the entrepreneurial success

The value discipline model proposed by Treacy and Wiersema (1993) was used by the research subject as the operating policy in the company’s operation process. This model refers to the principle of leading a company after comprehending the enterprise management system, operating process, organizational structure, and cultural differences. The guidelines for leading a business include product leadership, operational excellence, and customer intimacy.

#### Product leadership

The case company has positioned its products as being healthy, organic, and natural, with professional quality, and as the foundation of safe ingredients. The entrepreneur of the company has worked diligently to rebuild Taiwan’s food safety. In addition to performing innovative research and developing non-toxic and safe animal feed, the company has also realized the one-stop feed-to-breeding production model and strived to promote safe feeds. It sells natural feeds to farms and has its own farms, feed mixing plants, and feed factories. It also has further extended downstream to invest in animal breeding, and bred dragon sturgeon, weever, sweet fish, Ganoderma lucidum pigs, and other animals with its own high-quality feeds. The entrepreneur believes that good ingredients can only be cultivated with good food in a good environment, so a safe breeding cycle production mode has been adopted for the whole process.

In order to breed dragon sturgeon, the entrepreneur has carried out consistent pre-processing fishing inspections while raising the seedlings and controlled the quality of fish with a safe food material monitoring system, using only pure aquatic feeds made of natural raw materials. In the breeding of Ganoderma lucidum pigs, pregnant pigs are fed Ganoderma lucidum meal to enhance their immunity and then the piglets are also fed safe feed and Ganoderma when being raised. In addition to producing feed and cultivating healthy ingredients, the company has further stepped into the field of food processing by producing and processing healthy, organic, and natural food to make consumers feel at ease and eat healthily.

#### Operational excellence

In the early days of the company’s establishment, the pig raising industry was prosperous. There was a huge demand for feed in the market. The entrepreneur was successful when he started his business for the first time. With the identification of market development opportunities and the accumulation of past work experience, coupled with the contacts accumulated before the establishment of the company, the research subject experienced success in a short period of time. However, after the outbreak of hoof-and-mouth disease, the entrepreneur went against the flow and returned to the old business after selecting a factory. The extraordinary courage and perseverance of the entrepreneur was the main reason why the company could continue to operate. The entrepreneur used to be a veterinarian on a livestock farm, and he paid more attention to the safety and nutrition of the pigs’ food intake. He used his expertise to provide the pigs with safe feed in a perfect growth environment; the pigs were not only healthy but also had a relatively high growth rate. The company was also listed as a breeding demonstration farm, thereby reshaping the image and value of the pig industry.

After 2011, society began to pay attention to the issue of food safety. The entrepreneur has been providing good and safe feed raw materials as his corporate tenet since starting his entrepreneurial processes in the past. Driven by the food safety trend, the case company became committed to innovative research and development and proposed a one-stop breeding concept based on the entrepreneur’s belief that when animals eat healthily, people will have healthy food. He, therefore, integrated existing resources, and actively developed feeds that are safe for animals to eat, to withhold the standard from the source. After successfully developing safe feed products, the case company began to expand downstream. On the one hand, it provided feed to downstream manufacturers, and on the other hand, it also established its own farms, feed mixing plants, and feed factories. In addition, it also entered the field of food processing. Because it was familiar with consumers’ needs for food safety, it began producing safe and sound products.

#### Customer intimacy

The case company has vertically integrated resources and established a complete production and marketing system. The upstream is feed raw materials, the midstream is feed products, and the downstream is processed products made from animals that eat safe feeds. It also has its own brand, Ease Kitchen, which is used for promotion. In order to strengthen the concepts of safety and non-toxicity, it has cooperated with academic units to obtain traceable certificates for their products from scratch while promoting a series of safe production to consumers.

## Conclusion and discussion

### Conclusion

Entrepreneurship is the process of creating a business from nothing. Entrepreneurs must have the ability to identify opportunities as well as professional skills, resource acquisition, and business management capabilities. They are also affected by various external environmental factors such as customers or competitive pressure. Taiwan has been influenced by Confucian culture for more than 2,000 years. The concept of the five cardinal relationships suggests that people play different roles in a group, and that each role should perform its own responsibilities through the division of labor while cooperating to perform their duties well so the group can run smoothly. Being the leader of a group requires more responsibilities and obligations. The entrepreneur of the case company has upheld a persevering character, observed the possibility of starting a business after every failure, shouldered the heavy responsibility of entrepreneurship by him alone, and has gained the sweet fruit of his hard work in the end on the bumpy road. Entrepreneurial failure is not scary; what is scary for individuals is determining whether they are fully prepared to succeed when they start a business again.

For entrepreneurs, the prerequisite for entrepreneurial success is the ability to identify opportunities. Even during a crisis, as long as the situation can be clearly analyzed, turning points for the next entrepreneurship can be found. The accumulation of failure experiences in the three re-startups of the case company strengthened the Entrepreneur’s crisis management and risk assessment abilities. The entrepreneur of this research case chose to resume the old business when the hoof-and-mouth epidemic was the most serious, and he finally reorganized the resources of the enterprise, created a brand, and strictly controlled quality using innovative breeding methods to satisfy consumers’ demand for food safety. He succeeded because he identified the right time to enter the industry. Also, the availability of funds and manpower provided the biggest boosts for the entrepreneur’s start-ups. The entrepreneur relied on his ability to find market development potential and see opportunities, used his expertise and ability to select the industries, made a complete plan, and started his journey again.

**Figure 1:**
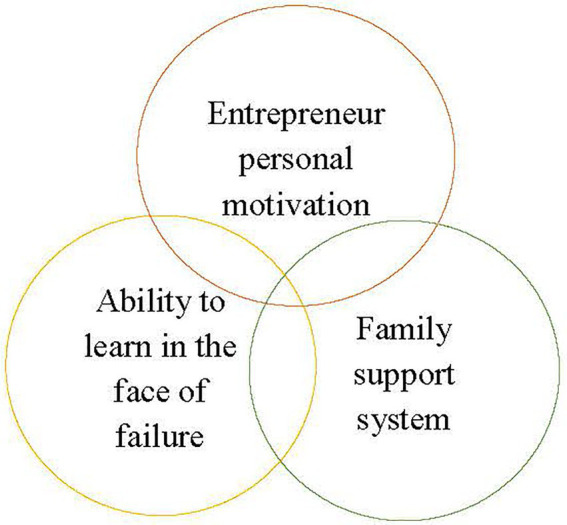
Reasons for re-entrepreneurship.

The entrepreneur of the case company fully explained his motivation and choice to start a business after his entrepreneurial failures. In addition to the above-mentioned attributions, the support of his family was another factor. The entrepreneur of the case company once said: “The support of the family is the biggest motivation for moving forward every time. Thinking of my family, I can overcome all difficulties.” As discussed in the work-family border theory by Clark (2000), the reasons for the continuous re-entrepreneurial attempts of the subject also included the relationship between work and family. Previous studies have shown that for entrepreneurs, a spouse is an important source of capital when starting a business (Matzek et al., 2010). In the work-family issue, the impact of family support on entrepreneurs is an important factor (Drummond et al., 2017; Lapierre et al., 2018). The perspective of Confucian role ethics shows that the family is the starting point for human beings to establish various relationships. The entrepreneur of the case company could always maintain his positive energy due to the encouragement and support of his family and relatives. Family support is rarely discussed in previous studies on the influence of entrepreneurial motivation after entrepreneurial failure, and the results of this study could fill this gap. The entrepreneur’s success was not accidental; family support made the entrepreneur more confident and encouraged him to persist in his entrepreneurial behavior. Figure 1 is a summary of the model of the reasons for re-entrepreneurship for this study.

### Discussion

This study conducted an in-depth interview with a serial entrepreneur who has experienced three entrepreneurial journeys regarding how he recovered from negative entrepreneurial experiences in the past and participated again in entrepreneurial activities. When entrepreneurs experience entrepreneurial failures, they may also have to bear debts and lawsuits, in addition to psychological blows, thus, returning to entrepreneurial activities will generally be more difficult than the first-time entrepreneurial attempt. If coupled with a lack of resources, they must rely on their stronger entrepreneurial motivation to drive them to resume entrepreneurship.

In addition to the ability to learn from failures (Plehn-Dujowich, 2010; Yamakawa et al., 2015; Soublière and Gehman, 2020) and recover from the cost of entrepreneurial failures (Eggers and Song, 2015; Nefzi, 2018), this study found that the family support system seemed to be an important factor in prompting entrepreneurs’ re-entrepreneurship, and this finding has also filled the theoretical gaps of and made functional explanations to entrepreneurs’ motivation for re-entrepreneurship after failures.

In practice, family support can also help entrepreneurs who have experienced entrepreneurial failures to recover as soon as possible and avoid being discouraged by their failure. Family support strengthens the individual’s resilience to failure and is seen as a valuable resource for overcoming failures and having self-recovery, thus, with family support, those who fail can re-start their entrepreneurial activities without fear. The results of this study contribute to understanding the motivation of entrepreneurs who have failed and then started again, provide a striking new vision, and add a larger cognitive perspective.

### Implications

Past entrepreneurial experience and the entrepreneur’s psychological recovery are necessary conditions for starting a new business. For entrepreneurs, a failure may be viewed as temporary and not equal to a personal failure, as an enterprise failure may not be caused by the entrepreneur’s personal mistakes. Sometimes external factors, such as a bad general environment, may cause enterprises to close down. Therefore, in addition to the ability to learn from failures, identifying an opportunity for re-entrepreneurship in an increasingly complex, highly uncertain, and challenging business environment is important, and the internal psychological adjustment of the individual is key to the success or failure of new entrepreneurship.

Therefore, an entrepreneur’s previous experience of failure in the process of starting a business results in a reduced failure rate when starting another business. Entrepreneurs accumulate the ability to judge things according to their failure experiences and will make more appropriate risk assessments going forward, which helps them cope with crises in future. These abilities all belong to the accumulation of experience at the personal level. The family support system is also a key influencing factor for an entrepreneur’s recovery from failure and continuous entrepreneurial behavior. Especially serial entrepreneurs who have experienced multiple entrepreneurial failures, they will regard family support as the most important resource both financially and psychologically, while family members may also actively help by providing support to help entrepreneurs recover as soon as possible after a failure. This study suggested that entrepreneurs follow the following methods in the entrepreneurial process. First, entrepreneurs must have insight into market trends and opportunities and set goals to achieve them courageously. Second, by identifying where they failed in the past, they should learn new skills to address such shortcomings in future, and apply them when they start another business. Third, entrepreneurs should make good use of the spiritual support and comfort brought by family resources, and regard them as important assets of entrepreneurship and an important backing to face every new challenge.

This study adopted typical case sampling to collect data through interviews; however, memories are often distorted and change over time (Baron and Ensley, 2006), thus, future research can explore the entrepreneur’s motivation for re-entrepreneurship in countries with similar cultures and values (e.g., China, Korea). Although the research results of this study provide a preliminary explanation for the psychological aspects of re-entrepreneurial motivation for entrepreneurs who have failed, the impact of a family support system on re-entrepreneurial motivation in different countries can also be applied in future research to enrich the conceptual gap of re-entrepreneurial motivation.

## Ethics statement

Ethical review and approval was not required for the study on human participants in accordance with the local legislation and institutional requirements. The participants provided their written informed consent to participate in this study. Written informed consent was obtained from the individual(s) for the publication of any potentially identifiable images or data included in this article.

## Author contributions

L-YP developed the main conceptual ideas and theoretical formalism and designed and performed the research framework. I-CT wrote the manuscript. S-HP carried out the implementation, collected the data, and wrote the draft. S-CC helped to supervise the project. All authors discussed the results, contributed to the final manuscript, and approved the submitted version.

## Conflict of interest

The authors declare that the research was conducted in the absence of any commercial or financial relationships that could be construed as a potential conflict of interest.

## Publisher’s note

All claims expressed in this article are solely those of the authors and do not necessarily represent those of their affiliated organizations, or those of the publisher, the editors and the reviewers. Any product that may be evaluated in this article, or claim that may be made by its manufacturer, is not guaranteed or endorsed by the publisher.
